# Fine-Tuning Roles of Endogenous Brain-Derived Neurotrophic Factor, TrkB and Sortilin in Colorectal Cancer Cell Survival

**DOI:** 10.1371/journal.pone.0025097

**Published:** 2011-09-26

**Authors:** Hussein Akil, Aurélie Perraud, Carole Mélin, Marie-Odile Jauberteau, Muriel Mathonnet

**Affiliations:** 1 Université de Limoges, Laboratoire EA 3842, Homéostasie Cellulaire et Pathologies, Faculté de Médecine et de Pharmacie, Limoges, France; 2 Université de Limoges, Institut Fédératif de Recherche 145 GEIST «Génomique, Environnement, Immunité, Santé et Thérapeutiques», Limoges, France; 3 CHU de Limoges, Service de chirurgie digestive générale et endocrinienne, Limoges, France; National Institute on Aging Intramural Research Program, United States of America

## Abstract

**Background:**

Neurotrophin receptors were initially identified in neural cells. They were recently detected in some cancers in association with invasiveness, but the function of these tyrosine kinase receptors was not previously investigated in colorectal cancer (CRC) cells.

**Methods and Findings:**

We report herein that human CRC cell lines synthesize the neural growth factor Brain-derived neurotrophic factor (BDNF) under stress conditions (serum starvation). In parallel, CRC cells expressed high- (TrkB) and low-affinity (p75^NTR^) receptors at the plasma membrane, whereas TrkA and TrkC, two other high affinity receptors for NGF and NT-3, respectively, were undetectable. We demonstrate that BDNF induced cell proliferation and had an anti-apoptotic effect mediated through TrkB, as assessed by K252a, a Trk pharmacologic inhibitor. It suppressed both cell proliferation and survival of CRC cells that do not express TrkA nor TrkC. In parallel to the increase of BDNF secretion, sortilin, a protein acting as a neurotrophin transporter as well as a co-receptor for p75^NTR^, was increased in the cytoplasm of primary and metastatic CRC cells, which suggests that sortilin could regulate neurotrophin transport in these cells. However, pro-BDNF, also detected in CRC cells, was co-expressed with p75^NTR^ at the cell membrane and co-localized with sortilin. In contrast to BDNF, exogenous pro-BDNF induced CRC apoptosis, which suggests that a counterbalance mechanism is involved in the control of CRC cell survival, through sortilin as the co-receptor for p75^NTR^, the high affinity receptor for pro-neurotrophins. Likewise, we show that BDNF and TrkB transcripts (and not p75^NTR^) are overexpressed in the patients' tumors by comparison with their adjacent normal tissues, notably in advanced stages of CRC.

**Conclusion:**

Taken together, these results highlight that BDNF and TrkB are essential for CRC cell growth and survival in vitro and in tumors. This autocrine loop could be of major importance to define new targeted therapies.

## Introduction

Brain-derived neurotrophic factor (BDNF), a member of the neurotrophin family, is known to play a critical role in the modulation of cell survival, differentiation and apoptosis in the nervous system [1].

BDNF signals through two types of cell surface receptors: the high-affinity tropomyosin-related kinase (Trk) receptor B (TrkB), a tyrosine kinase receptor and the low affinity receptor (p75^NTR^), as well as a death domain receptor belonging to the tumor necrosis factor (TNF) receptor family, a common receptor for all neurotrophins nerve growth factor (NGF), neurotrophin-3 (NT-3), and neurotrophin-4/5 (NT-4/5). Neurotrophins are synthesized as precursors (proneurotrophins) that are proteolytically cleaved to mature neurotrophins. Pro-BDNF cleavage can occur either intracellularly by the action of furin or proconvertase, or extracellularly by the action of plasmin, matrix metalloproteinase 7 (MMP-7) or MMP-9 [2]. Both mature BDNF and pro-BDNF are biologically active, with divergent roles that reflect differing receptor affinities: pro-BDNF displays higher affinities for p75^NTR^, whereas mature BDNF has greater affinities for TrkB.

BDNF binds the 145 TrkB full-length receptor and a truncated gp95TrkB variant that retains direct signaling activities [3] and increases specificity for BDNF [3], [4], [5]. While the Trk receptors are involved in most of the survival and growth properties of the neurotrophins, the functions of p75^NTR^, extensively studied in neurons, depends on neural cell type, the presence of ligand, and its association to a co-receptor. Indeed, p75^NTR^ associated with Trk co-receptor enhances [6] or suppress neurotrophin-mediated cell survival [7]. It was recently shown to be able to bind pro-BDNF and induce cell death when associated with sortilin (a member of the Vps10p-domain receptors family) [8], [9], [10].

Thus, regulation of pro-BDNF processing adds additional control over the balance between p75^NTR^ and TrkB engagement [11], [12]. The antiapoptotic function of BDNF is mediated though interaction with the high-affinity receptors 95 and 145TrkB [5], while pro-BDNF induces apoptosis via interaction with a receptor complex of p75^NTR^ and sortilin [10], [13].

Sortilin is expressed in several tissues, notably brain, spinal cord, heart, muscle, adipocytes [14] and B lymphocytes [8]. Sortilin was initially described in human neural cells as an intracellular transport protein for neurotrophins and proneurotrophins [15] and, recently, as a transporter of Trk neurotrophin receptors in neural cells [16]. Moreover, sortilin was also known as a co receptor (NTSR3) for a G protein-coupled receptor, the neurotensin receptor-1 (NTSR1) that is activated by neurotensin [17]. Neurotensin was initially shown to play a role in the growth and survival of colorectal cancer (CRC) cells, through its binding to this sortilin/NTSR1 complex [18].

BDNF has been implicated in the pathogenesis and prognosis of numerous human malignancies such as neuroblastomas [19], [20], medulloblastoma [21], prostate cancer [22], [23], lung cancer [24], pancreatic carcinoma [25], [26], [27], and hepatocellular carcinoma [28]. In CRC, an overexpression of BDNF [29] and TrkB [30] was recently reported in patients' tissues but no data deals with the function of BDNF as an autocrine loops in CRC cell survival. Since TrkB expression is associated to several cancers; the goal of this study was to define the conditions of endogenous secretion of BDNF and expression of neurotrophin receptors in CRC. Herein, we show that endogenous BDNF is secreted by CRC cells submitted to serum deprivation and induces cell survival through TrkB tyrosine kinase receptor that is expressed on the membrane of stressed cells. It is noteworthy that TrkB and BDNF expression was enhanced in patients' tumors especially in advanced stages. Collectively, these data point out the relevance of BDNF/TrkB pathway in the growth and potential invasiveness of CRC.

## Materials and Methods

### Cells and culture

Human CRC cell lines corresponding to different tumor stages were purchased from the American Type Culture Collection. WiDr [31] and SW480 [32] were primary CRC-derived cell lines, whereas the two other lines were derived from CRC metastases, in lymph node (SW620 deriving from the same patient as the SW480 line) [32] and ascitis (COLO 205) [33]. Under basal conditions, WiDr cells were maintained in MEM medium (Gibco) supplemented with 10% heat-inactivated Fetal Calf Serum (FCS) (Gibco), 1 mM sodium pyruvate (Gibco), 1% non essential amino acids (Gibco), 100 IU/ml penicillin and 100 mg/ml streptomycin (Gibco). SW480, SW620 and COLO 205 lines were cultured in RPMI medium (Gibco) supplemented with 10% FCS, 100 IU/ml penicillin and 100 mg/ml streptomycin, at 37°C under humidified atmosphere and 5% CO2. Cultured cells were stressed by 24 to 72-h serum starvation. The mouse antagonistic anti-BDNF monoclonal antibody (mAb) clone 35928.11 (10 µg/ml) was purchased from Calbiochem. Recombinant human BDNF (100 ng/ml), recombinant pro-BDNF (4 ng/ml), and K252a (200 nM) were purchased from Alomone labs.

### Patients and tissue samples

All patient-derived tissues were collected and archived, at the Tumorotheque of Limoges University Hospital, under protocols approved by the Institutional Review Board (AC N 2007-34, DC 2008-604 and 72-2011-18). Written informed consent was obtained by all subjects for this study. Tumor tissues were obtained from 20 patients, who underwent surgical removal of CRC in Limoges University Hospital between January 2006 and February 2007. Four adenocarcinoma patients per stage (according to the pTNM classification [34]) have been chosen for this study. Tissues from four patients with a benign colorectal disease, megadolichocolon, were used as controls.

### RT-PCR analysis

CRC cell lines were cultured in medium containing or not 10% FCS for 24 to 72-h. SV total RNA isolation system (Promega) was used to isolate total RNA from the cell lines as described in the manufacturer's instructions. The amount of RNA extracted was quantified by measuring the absorbance at 260 nm using the Nanodrop spectrophotometer ND-1000 (Labtech). The purity of the RNA was checked by the ratio DO260/DO280 nm between 1.83 and 2.00. The absence of RNA degradation was confirmed by electrophoresis on a 1.5% agarose gel containing ethidium bromide. Extraction of RNA from patients' tissues was performed as described [35]. First-strand cDNA synthesis was generated by using SuperScript III (Invitrogen). PCR was performed using Taq DNA polymerase (Invitrogen). Total RNA isolated from human neuroblastoma cell lines (IMR32, SH-SY5Y) and human erythromyeloblastoid leukemia K562 cells were used as positive controls. Non-reverse–transcribed samples (manufactured by omitting the reverse transcriptase) were run in parallel with the reverse-transcribed samples to exclude contamination by genomic DNA.

Primers were designed using Primer 3 (provided in the public domain by the Whitehead Institute for Biomedical Research/MIT Center for Genomic Research, Cambridge, MA, and available at http://www.wi.mit.edu). Designed primer pairs are listed, along with their expected product size and annealing temperatures in Table 1.

**Table 1:** Primers used in RT-PCR studies.

Name	Primers sequences	Amplified fragments	HT (°C)	3′ Location
BDNF				
F	TACTTTGGTTGCATGAAGGCTGCC	266 bp	58	596
R	ACTTGACTACTGAGCATCACCCTG			
TrkB145				
F	AGGGCAACCCGCCCACGGAA	571 bp	62.6	2860
R	GGATCGGTCTGGGGAAAAG			
TrkB95				
F	GTTTCATAAGATCCCACTGGA	261 bp	58	7111
R	TGCTGCTTAGCTGCCTGAGAG			
TrkA				
F	TCAACAAATGTGGACGGAGA	197 bp	58	1372
R	GTGGTGAACACAGGCATCAC			
TrkC				
F	ACTTCCGTCAGGGACACAAC	219 bp	58	1708
R	CCTCCCTCTGGAAATCCTTC			
p75^NTR^				
F	GTGGGACAGAGTCTGGGTGT	200 bp	60	3109
R	AAGGAGGGGAGGTGATAGGA			
Sortilin				
F	CTGGGTTTGGCACAATCTTT	199 bp	60	1161
R	CACCTTCCTCCTTGGTCAAA			
Sortilin EC				
F	TCCTGGGTTGGAGATAGCAC	231 bp	58	392
R	TTCCTCCAGACACCTCTGCT			
Sortilin IC				
F	GCTGGTCACAGTCGTAGCAG	150 bp	58	2350
R	TTAGTGTGGGAGGCTGTGTC			
GAPDH				
F	GGGTGGAATCATATTGGAACATG	150 bp	58	2199
R	GTCGGAGTCAACGGATTTGG			

F, forward; R, reverse; HT, hybridation temperature.

### Sequencing

After extraction of PCR products with Rapid PCR Purification Systems (MARLIGEN bioscience), according to the manufacturer's instructions, PCR products were directly sequenced using the BigDye® Terminator v1.1 Cycle Sequencing Kit (Applied Biosystems) in a GeneAmp® PCRSystem 2700 thermocycler (Applied Biosystems). The fragments were purified by isopropanol precipitation. The sequencing gel was run on an automated laser fluorescent DNA sequencer ABI Prism® 3130×l Genetic Analyser (Applied Biosystems) and homologies were checked, using Finch TV, after blasting with the BDNF, TrkB145, TrkB95, p75^NTR^, and sortilin GenBank sequences (NM_001143816, NM_001018064, NM_001007097, NM_002507, and NM_002959, respectively).

### Western blot

Mouse anti-BDNF (1 µg/ml) and mouse anti-TrkB (1 µg/ml) antibodies (Abs) were purchased from R&D Systems, rabbit anti-P75^NTR^ (1 µg/ml), goat anti-sortilin (1 µg/ml) and rabbit anti-phospho-Akt (Ser 473; 1 µg/ml) Abs from Santa Cruz Biotechnology, rabbit anti-TrkA (1∶1000), rabbit anti-TrkC (1∶1000) and mouse anti-Akt (1∶2000) Abs from Cell Signaling Technology. Subconfluent cell lines cultures were lysed (5 minutes on ice) in the culture flasks by 1× Cell lysis buffer (Cell Signaling Technology) containing 1 mM PMSF (Sigma-Aldrich). The suspension was sonicated for 1 min (a pulsation of 40 Hz every 20 sec) to degrade chromosomal DNA and centrifuged at 14,000 g for 10 minutes. Supernatants were collected and protein concentration was determined using Bradford protein concentration assay (Sigma). SDS-PAGE was performed and the proteins were electro-blotted onto PVDF membranes (BioTrace™). After one hour of incubation at room temperature in blocking solution (5% non-fat dry milk in PBS), the membranes were exposed to the specific primary Abs in blocking solution overnight at 4°C. Then, membranes were washed thrice for 5 min with TBS/0.1% Tween-20 and the immunoreactions were detected by horseradish peroxidase-conjugated secondary Ab to mouse, rabbit, or goat Ig (Dakocytomation) diluted at 1∶2000 in blocking solution for 1 h at room temperature. After washing, visualization of immunocomplexes was accomplished using the Immobilon Western Chemiluminescent HRP Substrate (Millipore). Protein-loading control was performed with anti-Actin Ab (Cell Signaling Technology). Western blots were scanned using a bio-imaging system (Genesnap; Genetool; Syngene). Densitometric analyses were performed using an IMAGEJ software program (National Institutes of Health, Bethesda, MD, USA http://rsb.info.nih.gov/ij/). Protein expression was determined in relative units in reference to actin expression.

### Immunofluorescence

Cells grown on 12-mm coverslips were fixed with 4% PFA at room temperature for 30 min, and permeabilized or not with 0.1% Triton X-100. Nonspecific binding was blocked by 30 min incubation with PBS-2% BSA at room temperature. Coverslips were then incubated overnight at 4°C in blocking solution with the primary Ab. The following Abs were used: rabbit anti-BDNF Ab (1 µg/ml; Santa Cruz Biotechnology), rabbit anti-pro-BDNF Ab (8 µg/ml; Alomone Labs), mouse anti-TrkB Ab (2.5 µg/ml; R&D Systems), rabbit anti-p75^NTR^ (2 µg/ml; Santa Cruz Biotechnology) and goat anti-sortilin Ab (1 µg/ml; Santa Cruz Biotechnology). Cells were washed 3 times in PBS, and incubated for 2 h at room temperature with Alexa Fluor-conjugated secondary Abs (Invitrogen) diluted 1∶5000 in PBS. After 3 washes in PBS, nuclei were stained for 5 min with DAPI. After intensive washes, coverslips were inverted on slides and mounted with Dako Fluorescent Mounting Medium (Dakocytomation). Negative controls were cells incubated with irrelevant normal rabbit, mouse, or goat IgG (Sigma). Pictures were taken using a confocal microscope (Carl Zeiss, LSM 510); surface plots of fluorescence data were generated with IMAGEJ software program.

### ELISA

After a 24-h, 48-h and 72-h culture, the samples of supernatant from cell lines were collected and stored at −80°C until the day of assay. Immunoassays Systems (Promega), specific for BDNF were performed according to the manufacturer's instructions. Results were expressed as mean ± SEM (pg/mL). At least three independent experiments were performed for each experimental condition, each with measurements in triplicate.

### Cell proliferation assays

Cell proliferation was measured using the Click-it EdU Alexa Fluor 488 Flow cytometry assay (Invitrogen), according to the manufacturer's instructions. Briefly, cells (2×10^5^ per well) were incubated overnight on twelve-well plates before treatment, then cultured for 24-h in serum-free medium with or without exogenous BDNF, K252a or both BDNF and K252a added simultaneously. Proliferation values were measured using a BD LSRFortessa flow cytometry (BD Biosciences). Experiments were performed in triplicate, and three sets of 50,000 cells were collected for every condition. The data were acquired and analyzed by the BD FACSDiva 6.0 software (BD Biosciences). Each experiment was repeated at least thrice.

### Apoptosis assay

Apoptosis was measured by the detection of cytoplasmic soluble nucleosomes using a calorimetric assay, Cell Death Detection ELISAPLUS kit (Roche Molecular Diagnostic) according to the manufacturer's instructions. Absorbance values were measured at 405–490 nm dual wavelengths. The absorbance obtained in controls was normalized to a value of 1, as previously described [7]. All the experiments were performed in triplicate and repeated at least three times.

### Statistical analysis

Statistical significance was determined by a one-way analysis of variances (ANOVA) with statview 5.0 software (Abacus Concepts). *P* values<0.05 were considered significant. Mean and SEM values were obtained from at least 3 independent experiments.

## Results

### ColoRectal Cancer cell lines express TrkB and p75^NTR^ but not TrkA or TrkC receptors

**Figure 1:**
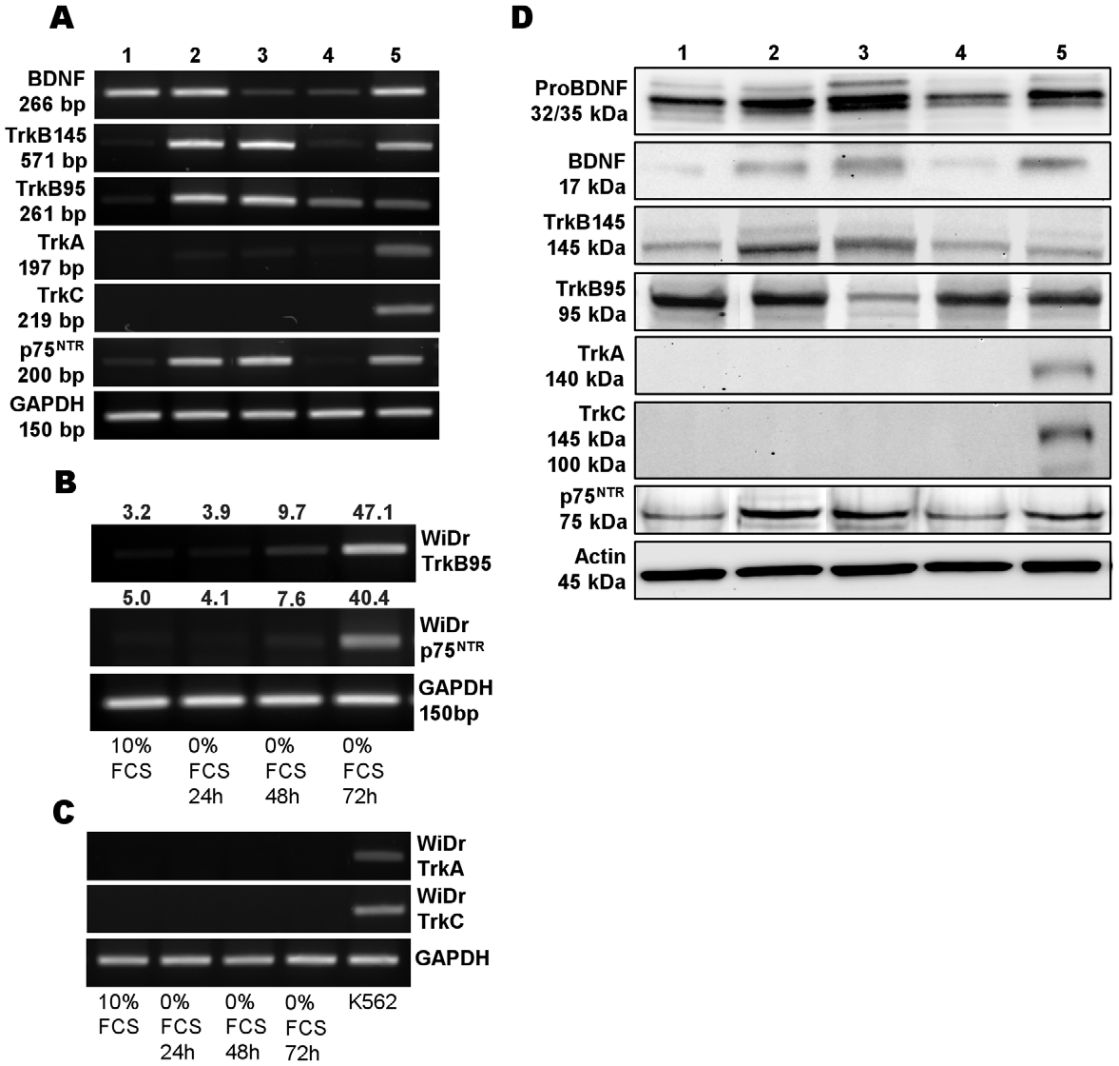
Expression of BDNF and its receptors in human CRC cell lines. (A): RT-PCR of BDNF, its high (TrkB) and low (p75^NTR^) affinity receptors, TrkA, and TrkC from cells cultured in basal (10% FCS medium), WiDr (lane: 1), SW480 (lane: 2), SW620 (lane: 3), COLO 205 (lane: 4). GAPDH mRNA levels was used as an internal control. Positive control (lane 5) was the neuroblastoma cell line (IMR32) for BDNF, TrkB and p75^NTR^; and the erythromyeloblastoid leukemia cells (K562) for TrkA and C. A representative result of at least three to five independent experiments. (B) Comparison of TrkB95 and p75^NTR^ on total RNA extracted from WiDr cells cultured in 10% FCS or after 24 to 72 h serum deprivation (0% FCS). TrkB95 and p75^NTR^ mRNA/GAPDH mRNA quantification of band intensities evaluated by densitometry are shown above lanes and expressed in arbitrary units (mean of three independent experiments). (C) Same experiment: RT-PCR of TrkA and TrkC on total RNA extracted from WiDr cells cultured under basal culture conditions (10% FCS) and after 24–72 h of serum starvation in comparison to positive control (K562) (D) Expression of pro-BDNF and BDNF, full length TrkB 145 and truncated TrkB 95 and p75^NTR^ proteins in CRC cell lines cultured in 10% FCS. TrkA and TrkC were not detected. Actin was used as loading protein control. WiDr (lane: 1), SW480 (lane: 2), SW620 (lane: 3), COLO 205 (lane: 4). Positive control (lane: 5) were IMR32 cells for BDNF, pro-BDNF, TrkB and p75^NTR^ and K562 cells or TrkA and TrkC. A representative result of at least three independent experiments.

TrkB and p75^NTR^ expressions were detected in CRC cell lines under basal (10% FCS) culture conditions, both at mRNA (Figure 1A) and protein (Figure 1D) levels with some differences depending on cell lines. Whereas the full length TrkB (TrkB145) and p75^NTR^ transcripts were predominant in a primary (SW480) and a metastasis (SW620) line (Figure 1A) (two cell lines isolated from the same patient), the most strongly expressed transcripts were those of the truncated isoform (TrkB95) in all cell lines (Figure 1A) except for WiDr cells that expressed TrkB95 and p75^NTR^ only after a 48-h serum deprivation (Figure 1B). TrkB and p75^NTR^ sequencing after agarose elution gels validated these results. However, TrkA and TrkC transcripts (Figure 1C) as well as proteins receptors (Figure 1D) were not detected, whatever culture conditions, in contrast to the erythromyeloblastoid leukemia K562 cell line known to express these neurotrophin receptors [36].

### CRC cells produce endogenous BDNF

**Figure 2:**
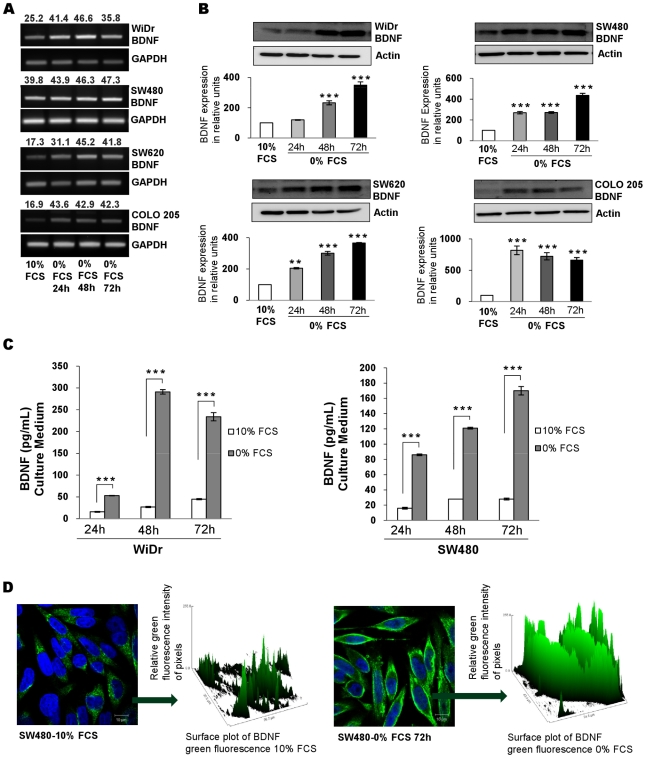
Comparison of BDNF production in CRC cells cultured with 10% or without FCS (0% FCS). (A) BDNF production assessed by RT-PCR of total RNA extracted from cells cultured in basal condition (10% FCS) and for 24 to 72 h of serum deprivation. Quantification of band intensities is shown as above (mean of three independent experiments). (B) BDNF expression by western blotting (in reference to actin) in total cellular protein extracted from cell lines cultured under basal condition (10% FCS) and after 24–72 h of serum deprivation (0% FCS). According to densitometric analyses, quantification showed a significant increased expression of BDNF in cultured cells. Histograms are means ± SEM of at least three independent experiments. **, *p*<0.01; ***, *p*<0.001, in comparison with basal culture conditions. (C) Secretion of BDNF assessed by ELISA in supernatant of the WiDr and SW480 cell lines under basal condition (10% FCS) and after 24–72 h of serum deprivation (0% FCS). Results are expressed as the mean ±SEM of triplicates from three different experiments. ***, *p*<0.001, when compared with basal culture condition. (D) Comparison of BDNF expression by SW480 cells cultured in 10% FCS and after 72-h serum deprivation. Confocal microscopy with anti-BDNF Ab and Alexa fluor-488 conjugate (green) and nuclei counter stained with the blue-fluorescent DNA stain DAPI. Relative quantification was assessed by green fluorescence surface plot. Images were representative for at least three independent experiments. Scale bars, 10 µm. Similar results were observed with the three other lines (data not shown).

Since CRC expressed TrkB receptors, we searched for an endogenous production of BDNF, a TrkB ligand. BDNF mRNA was detected in all studied cell lines under basal (10% FCS) conditions, predominantly in the two primary CRC lines (WiDr and SW480). Interestingly, following serum starvation for 24 to 72 h, BDNF mRNA levels (Figure 2A), as well as mature BDNF protein (17 kDa) detected by Western blot in cell lysates, were enhanced in the four CRC cells (Figure 2B), as shown by densitometry values (BDNF/GAPDH ratios for mRNA and BDNF/Actin ratios for proteins) (Figure 2A, B). In addition, BDNF secretion was detected by ELISA in culture supernatant of CRC cell lines. BDNF release was significantly increased by serum starvation in the two primary WiDr and SW480 CRC cell lines (Figure 2C and Table 2) whereas, it was no significantly enhanced in metastatic SW620 and COLO 205 CRC cell lines (Table 3). Serum deprivation led to an increase of BDNF levels in the cytoplasm of these four cell lines as shown for SW480 in Figure 2D. Quantifying the green fluorescence intensity in every culture conditions confirmed the findings from the fluorescence images (Figure 2D) and reinforced the results obtained by Western blotting.

**Table 2:** BDNF release and effect of exogenous BDNF, pro-BDNF, TrkB inhibitor (K252a) and neutralizing anti-BDNF on apoptosis and proliferation in primary CRC cell lines.

	WiDr		
	BDNF Release		
	24 H	48 H	72 H
**10% FCS**	16±0.98	27±1.23	45±1.28
**0% FCS**	53±0.57***	291±5.03***	234±9.45***

	EDU	ECD		
	24 H	24 H	48 H	72 H
**10% FCS**	1±0.03	1±0.03	1±0.07	1±0.03
**10%+BDNF**	1.12±0.04	1±0.06	0.97±0.08	0.98±0.02
**0% FCS**	0.47±0.06	1.19±0.03	1.3±0.02	1.38±0.04
**0%+BDNF**	0.75±0.04**	1.01±0.03**	1.07±0.02***	1.09±0.03**
**0%+BDNF/K252a**	0.003±0.001***	1.1±0.02*	1.4±0.04	1.6±0.07**
**0%+K252a**	0.015±0.001***	1.34±0.02**	1.63±0.04***	1.8±0.05***
**0%+Anti-BDNF**	nd	1.62±0.04***	nd	nd
**0%+Pro-BDNF**	nd	1.3±0.02**	nd	nd

	SW480		
	BDNF Release		
	24 H	48 H	72 H
**10% FCS**	16±1.15	28±1	28±1.2
**0% FCS**	86±1.5***	121±1.2***	170±5.56***

	EDU	ECD		
	24 H	24 H	48 H	72 H
**10% FCS**	1±0.07	1±0.06	1±0.04	1±0.05
**10%+BDNF**	1.09±0.06	1.06±0.05	0.95±0.08	1±0.01
**0% FCS**	0.46±0.07	1.36±0.05	1.29±0.04	1.24±0.03
**0%+BDNF**	0.81±0.03***	1.04±0.02***	0.93±0.03***	0.84±0.05***
**0%+BDNF/K252a**	0.05±0.006***	1.45±0.02	1.43±0.07	1.41±0.05*
**0%+K252a**	0.04±0.005***	1.73±0.06***	1.6±0.03**	1.73±0.04***
**0%+Anti-BDNF**	nd	1.82±0.04***	nd	nd
**0%+Pro-BDNF**	nd	1.42±0.02***	nd	nd

Results are expressed as relative units of proliferating cells (EDU) or apoptotic ratios of soluble nucleosomes detected by ELISA Cell Death (ECD) after 24 to 72-hours (H) of serum deprivation (0% FCS). Mean ± SEM of at least three independent experiments.

*, *p*<0.05;

**, *p*<0.01;

***, *p*<0.001, compared with serum-free condition alone (0% FCS).

**Table 3:** BDNF release and effect of exogenous BDNF, pro-BDNF, TrkB inhibitor (K252a) and neutralizing anti-BDNF on apoptosis and proliferation in metastatic CRC cell lines.

	SW620		
	BDNF Release		
	24 H	48 H	72 H
**10% FCS**	9±0.57	11±1	15±2.5
**0% FCS**	11±0.5	13±1	17±1

	EDU	ECD		
	24 H	24 H	48 H	72 H
**10% FCS**	1±0.05	1±0.06	1±0.09	1±0.08
**10%+BDNF**	0.94±0.045	1.04±0.08	1.03±0.08	1±0.08
**0% FCS**	0.34±0.035	1.38±0.03	1.33±0.01	1.34±0.06
**0%+BDNF**	0.83±0.06***	0.8±0.12***	0.67±0.02***	0.57±0.03***
**0%+BDNF/K252a**	0.18±0.006*	1.7±0.06*	1.9±0.02***	2.1±0.02***
**0%+K252a**	0.08±0.012**	1.96±0.06***	2.4±0.05***	2.8±0.09***
**0%+Anti-BDNF**	nd	1.28±0.04**	nd	nd
**0%+Pro-BDNF**	nd	1.22±0.02**	nd	nd

	COLO 205		
	BDNF Release		
	24 H	48 H	72 H
**10% FCS**	9±1	10±1.1	10±0.5
**0% FCS**	10±1	11±0.5	15±0.4

	EDU	ECD		
	24 H	24 H	48 H	72 H
**10% FCS**	1±0.088	1±0.06	1±0.03	1±0.04
**10%+BDNF**	0.86±0.03	1±0.07	1±0.03	1±0.05
**0% FCS**	0.3±0.012	1.08±0.04	1.08±0.002	1.02±0.007
**0%+BDNF**	0.64±0.016***	0.6±0.04***	0.73±0.01***	0.68±0.03***
**0%+BDNF/K252a**	0.38±0.012*	1.24±0.05*	1.8±0.03***	1.8±0.04***
**0%+K252a**	0.17±0.01***	1.65±0.04***	2.5±0.1***	2.3±0.06***
**0%+Anti-BDNF**	nd	1.22±0.03**	nd	nd
**0%+Pro-BDNF**	nd	1.3±0.04**	nd	nd

Results are expressed as relative units of proliferating cells (EDU) or apoptotic ratios of soluble nucleosomes detected by ELISA Cell Death (ECD) after 24 to 72 hours (H) of serum deprivation (0% FCS). Mean ± SEM of at least three independent experiments.

*, *p*<0.05;

**, *p*<0.01;

***, *p*<0.001, compared with serum-free condition alone (0% FCS).

### Serum starvation induces membranous expression of TrkB and its colocalization with BDNF

**Figure 3:**
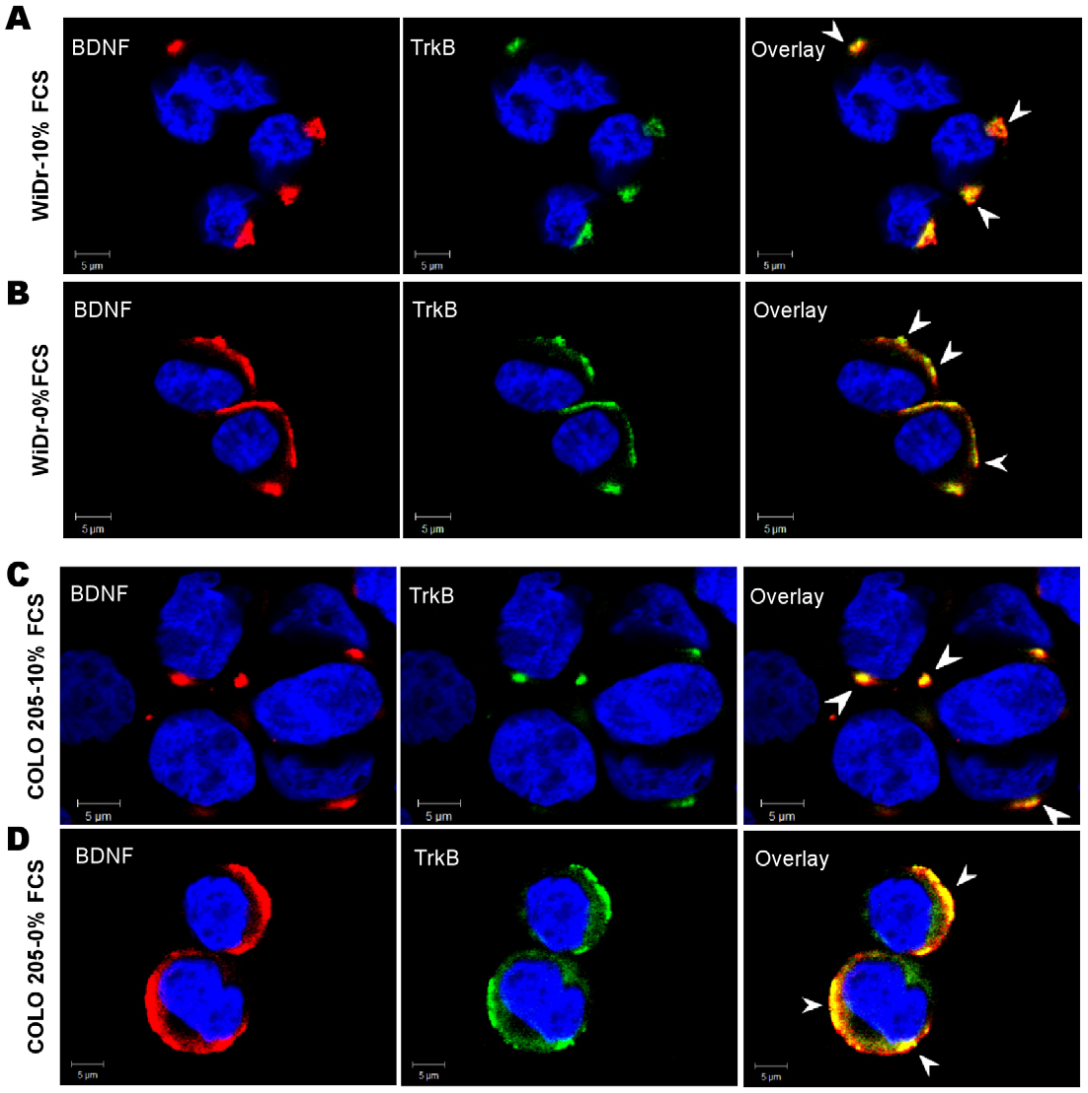
Membranous and cytoplasmic expression of BDNF and TrkB depending on culture conditions. Confocal microscopy of WiDr (A, B) and COLO 205 (C, D) cells, stained with an anti-BDNF Ab (red), anti-TrkB mAb (green) or both (overlay) cultured with 10% FCS (A, C) or after 24-h serum deprivation (B, D). Under basal culture conditions (10% FCS), TrkB and BDNF were sequestered in the cytoplasm (arrows) in WiDr (A) and COLO 205 (C) cells. The same staining patterns were obtained with the two other cell lines (data not shown). After serum starvation relocation to the cell membrane and colocalization of TrkB and BDNF (yellow in merged, arrows) were detected in WiDr (B) and COLO 205 (D). Images were representative for at least three to five independent experiments.

The finding that endogenous BDNF is secreted under serum starvation conditions led us to search for the expression of its high affinity receptor. In basal (FCS-containing) cultures (Figure 3A, C), the high affinity receptor TrkB and its ligand BDNF were sequestered in all CRC cell lines. A 24-h serum starvation induced a relocation of TrkB to the cell membrane (Figure 3B, D). Interestingly, a colocalization of TrkB and BDNF on the membrane was detected in all studied cell lines and reached a maximum at 72 h of deprivation, as shown for WiDr and COLO 205 cells (Figure 3B, D). Similar staining patterns were obtained for SW480 and SW620 (data not shown). The coexpression at the membrane of both TrkB and endogenous BDNF suggests that BDNF and TrkB could be implicated in an autocrine loop in stressed CRC cells.

### BDNF promotes proliferation and survival of CRC cell lines through TrkB

**Figure 4:**
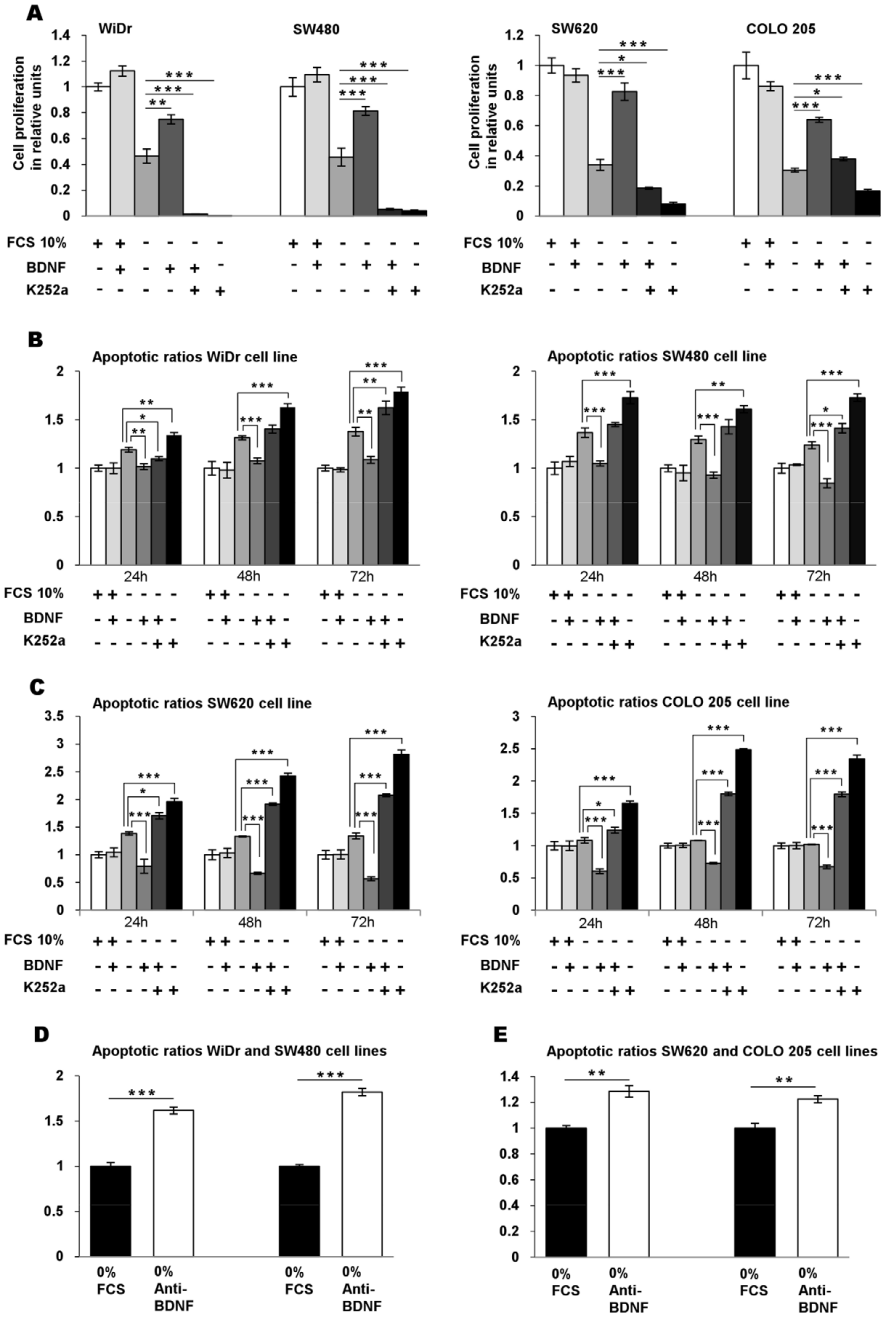
BDNF-TrkB promotes the cell survival of CRC cell lines. (A) Role of endogenous BDNF and its receptor TrkB on CRC cell proliferation: effects of exogenous BDNF and suppressing endogenous TrkB receptor on cell proliferation. The four cell lines were cultured for 24 h in FCS-free medium (FCS 10%, −) in the presence of exogenous BDNF (+), K252a (+) alone or in combination. Cell proliferation was determined by flow cytometry analysis using EdU Alexa Fluor 488. The data are presented as histograms of proliferating cells in relative units ± SEM of five independent experiments. *, *p*<0.05; **, *p*<0.01; ***, *p*<0.001, when compared with serum-free medium. (B, C) Effects of exogenous BDNF and suppressing endogenous TrkB receptor on cell survival. Apoptotic ratios of soluble nucleosomes were detected by ELISA Cell for WiDr, SW480, SW620, and COLO 205 induced by serum deprivation alone (FCS 10%, −) or in association either with exogenous BDNF (+), or with K252a (+), during 24–72 h of serum deprivation. Histograms, mean ratio of apoptotic cells ± SEM of at least three independent experiments. *, *p*<0.05; **, *p*<0.01; ***, *p*<0.001, when compared with serum-free condition alone. (D, E) apoptotic ratio after 24 h serum deprivation alone (0% FCS) or with combination with a neutralizing anti-BDNF mAb (0% anti-BDNF). Histograms, mean ratio of apoptotic cells ± SEM of three independent experiments. *, *p*<0.05; **, *p*<0.01; ***, *p*<0.001, when compared with serum-free condition alone.

To determine the function of this ligand-receptor system in the proliferation and survival of WiDr, SW480, SW620 and COLO 205 CRC cells, proliferation and apoptosis assays were performed with exogenous BDNF either in FCS-free or in 10% FCS-containing cultures. After a 24-h culture in serum-free medium, exogenous BDNF significantly increased the proliferation of all studied cell lines, in contrast to the absence of effect in the presence of 10% FCS (Figure 4A and Table 2, 3). Since TrkB was expressed at the surface of stressed cells, we hypothesized its possible role in the cell proliferation under such conditions. Indeed, the addition of K252a, a Trk inhibitor [37], suppressed the proliferative effect of exogenous BDNF in serum-free cultures in all studied cell lines (Figure 4A) which suggests that endogenous BDNF was implicated in cell growth through TrkB (Fig. 4A and Table 2, 3). To further evaluate the role of TrkB and BDNF in stressed CRC cell survival, apoptosis was evaluated by soluble nucleosome cytoplasmic levels in cultures with and without exogenous BDNF or K252a. After a 24-h serum starvation, WiDr, SW480, SW620, and COLO 205 cells stimulated with exogenous BDNF had significantly decreased apoptotic ratios, whereas no significant effect was observed on cells maintained in normal medium (Figure 4B, C and Table 2, 3). Moreover, 200 nM K252a increased the apoptotic ratios of WiDr, SW480, SW620, and COLO 205 (Figure 4B, C and Table 2, 3). The results obtained after 48 and 72-h serum starvation confirmed these data (Fig. 4B, C), suggesting that the proliferation of CRC cells might be mediated by a BDNF/TrkB signaling pathway.

**Figure 5:**
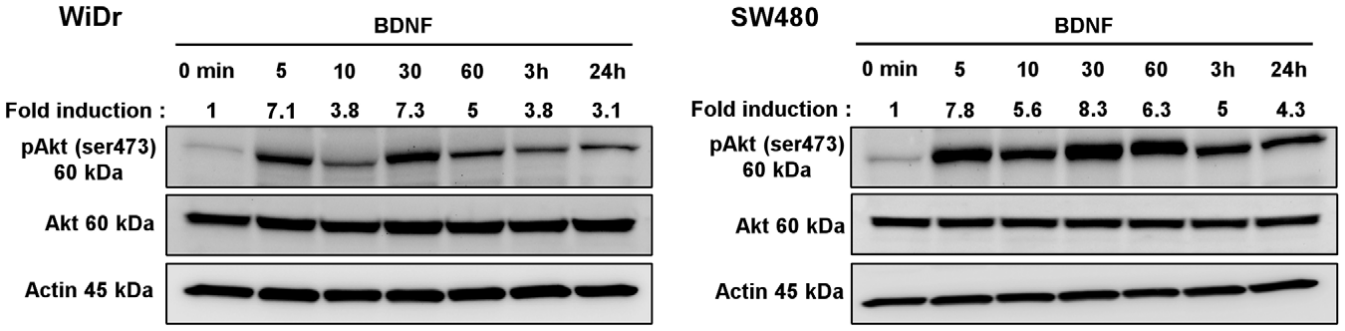
BDNF triggers phosphorylation of Akt in serum-starved CRC cell lines. The ability of BDNF to activate PI3-kinase/Akt signaling pathway in CRC cells was assessed using antibodies specific to Akt and phospho-Akt (pAkt). WiDr and SW480 cells were serum starved for 16 h. The cells were then exposed to BDNF (100 ng/ml) and harvested at different times, for 5 minutes (min) to 24 hours (h). Thirty µg of protein lysates was analyzed for pAkt (Ser473) and total Akt by western blot analysis. The density of each pAkt band was corrected for variance in loading, using the density of the corresponding total Akt. The fold induction was evaluated as the ratio of phosphorylated Akt protein densities between control (0 min) and treated cells. A representative result of at least three independent experiments.

To define the signal transduction pathway induced by BDNF/TrkB activation, we searched for Akt phosphorylation in two CRC cell lines following BDNF treatment. Indeed, western blotting revealed that the exposure of WiDr or SW480 to BDNF after a 16-hour serum deprivation, induced Akt phosphorylation (Ser 473) after 5 minutes, reached maximum at 30 minutes (7 to 8-fold increase) and yet detected after 24 hours (3 to 4-fold increase) (Figure 5).

We therefore determined apoptotic levels of the four cell lines in the presence of a neutralizing anti-BDNF mAb [38]. This mAb indeed increased the apoptosis of primary CRC lines: WiDr (Figure 4D, left and Table 2), SW480 (Figure 4D, right and Table 2) and metastatic lines, SW620 (Figure 4E, left and Table 3) and COLO 205 (Figure 4E, right and Table 3).

Altogether, these data suggest that endogenous BDNF is implicated in CRC cell survival in serum-free cultures via TrkB through an autocrine loop. This led to study the role of sortilin in BDNF traffic.

### Sortilin, a BDNF trafficking protein, is expressed by CRC cell lines

**Figure 6:**
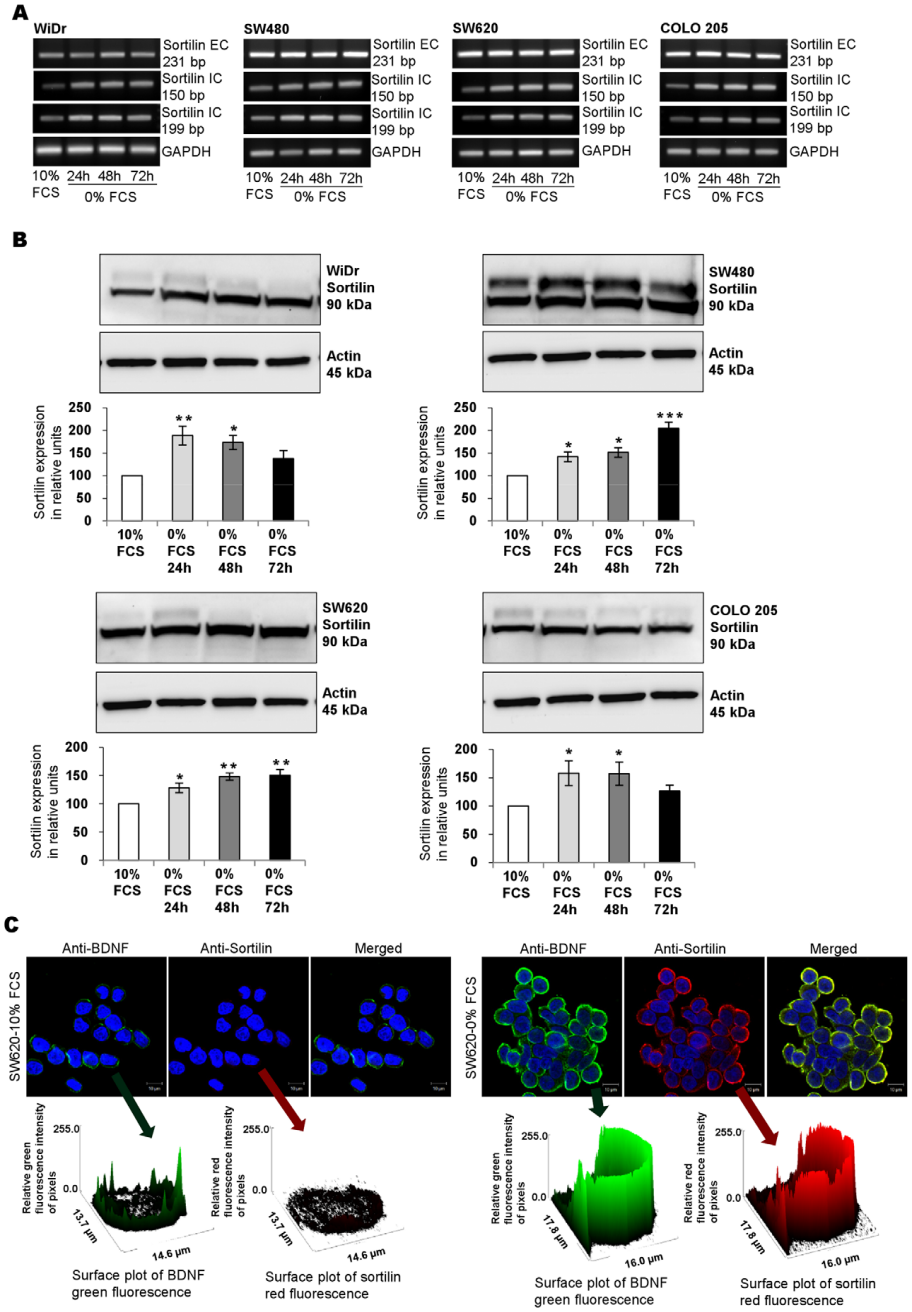
Sortilin expression by CRC cell lines. (A) Sortilin detection by RT-PCR of total RNA extracted from cells cultured in 10% FCS and after 24–72 h serum starvation. Expression was controlled with specific primers for its extracellular (Sortilin EC) and intracellular (Sortilin IC) parts. A Representative result from at least three independent experiments. (B) Assessment by western blotting of sortilin expression (in reference to actin) in total cellular protein extracted from studied cell lines cultured under basal condition and after 24–72 h of serum deprivation. According to densitometric analyses, quantification showed a significant increased expression of sortilin in cultured cells. Histograms are means ± SEM of at least three independent experiments. *, *p*<0.05; **, *p*<0.01; ***, *p*<0.001, when compared with basal culture condition (10% FCS). (C) Confocal microscopy of SW620 cells stained with an anti-BDNF Ab (green) and an anti-Sortilin Ab (red) and double staining (merged) in basal culture condition and after 24 h serum starvation. Relative quantification was assessed by green and red fluorescence surface plot. Images were representative for at least three independent experiments. Scale bars, 10 µm. Similar results were observed with the three other lines (data not shown).

The primers used in the study of sortilin transcripts recognized the intracellular part of the protein (sortilin IC involved in neurotrophin trafficking) and the extracellular part (sortilin EC involved in its receptor properties). Sortilin transcript (Figure 6A) and protein (Figure 6B) were detected in all studied cell lines. By comparison with cells cultured in 10% FCS, a 24 to 72-h serum deprivation enhanced sortilin expression as detected on immunoblots of WiDr, SW480, SW620 and COLO 205 extracts (Figure 6B). Sortilin is known to exist under two different states of glycosylation in CRC cells. Indeed, it was detected as a doublet in the SW480 cell line (Figure 6B), but barely in others (WiDr, Colo205) with an expression variable between cell lines and cultures (Figure 6B). Sortilin was detected in all CRC cell lines by confocal microscopy also, as shown for SW620 (Figure 6C). Double-staining for sortilin and BDNF showed a striking colocalization with increased fluorescence intensities after 24-h serum starvation (Figure 6C). Quantifying the green (BDNF) and red (sortilin) fluorescence intensities in each culture conditions confirmed the findings from the fluorescence images (Figure 6C). Similar staining patterns were observed with WiDr, SW620, and COLO 205 cell lines under the same conditions (data not shown). Altogether, our results are in agreement with the hypothesis that sortilin could exert the function of transporter for BDNF.

### Pro-BDNF induces CRC cell apoptosis through activation of a p75^NTR^-sortilin receptor complex

**Figure 7:**
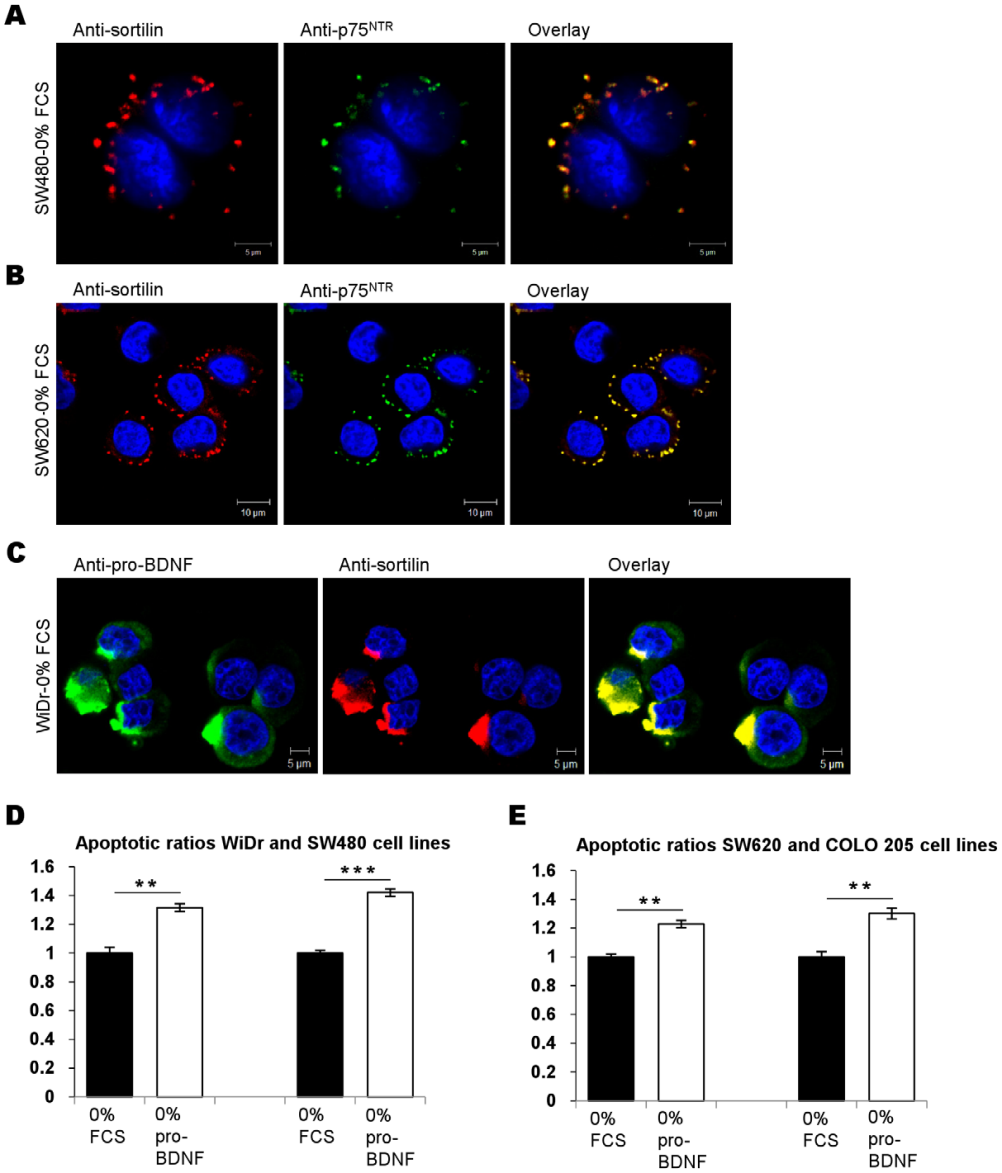
Relationship between pro-BDNF, sortilin, p75^NTR^ and apoptosis. (A, B) Sortilin as a coreceptor of p75^NTR^. Double staining (yellow) of sortilin (red) and p75^NTR^ (green) in SW480 cells (A) and SW620 (B) after 24 h of serum deprivation. (C) Colocalization of pro-BDNF and sortilin. Confocal microscopy study of a WiDr cells stained with an anti-pro-BDNF Ab (green) and an anti-sortilin Ab (red), and double staining (yellow) after 24 h of serum deprivation. (D, E) apoptotic ratios after 24 h serum deprivation alone (0% FCS) or combined with recombinant Pro-BDNF (0% Pro-BDNF). Histograms, mean ratio of apoptotic cells ± SEM of three independent experiments. *, *p*<0.05; **, *p*<0.01; ***, *p*<0.001, when compared with serum-free condition alone (0% FCS).

The immature form of BDNF, pro-BDNF was also detected by Western blotting in all CRC cell lines as a 32–34 kDa protein doublet (Figure 1D). In addition to its role of precursor leading to mature BDNF, a specific and opposite function is related to its high affinity binding to p75^NTR^, a death domain receptor, as evidenced in neurons and B lymphocytes but not yet in CRC cells. We thus examined the functional effect of exogenous pro-BDNF on the four CRC cell lines maintained in serum-free medium. Apoptotic ratios significantly increased in all CRC lines, especially in the two primary lines (Figure 7D, E and Table 2, 3). We speculated that pro-BDNF exerts pro-apoptotic activity through the recruitment of sortilin by p75^NTR^ as known in neurons [10]. To address this hypothesis, we evaluated the coexpression of sortilin and p75^NTR^ by confocal microscopy. As illustrated with SW480 and SW620 lines, a clear-cut colocalization and punctiform polarization of p75^NTR^ and sortilin in the cell membrane was observed in all CRC cell lines (Figure 7A, B). On the other hand, pro-BDNF was also colocalized with sortilin as detected for WiDr (Figure 7C) as for the three other cell lines (data not shown). These findings suggest that pro-BDNF and the complex p75^NTR^/sortilin could counterbalance the autocrine survival TrkB/mature BDNF loops in CRC cell lines.

### BDNF and its receptors are highly expressed *in vivo*

**Figure 8:**
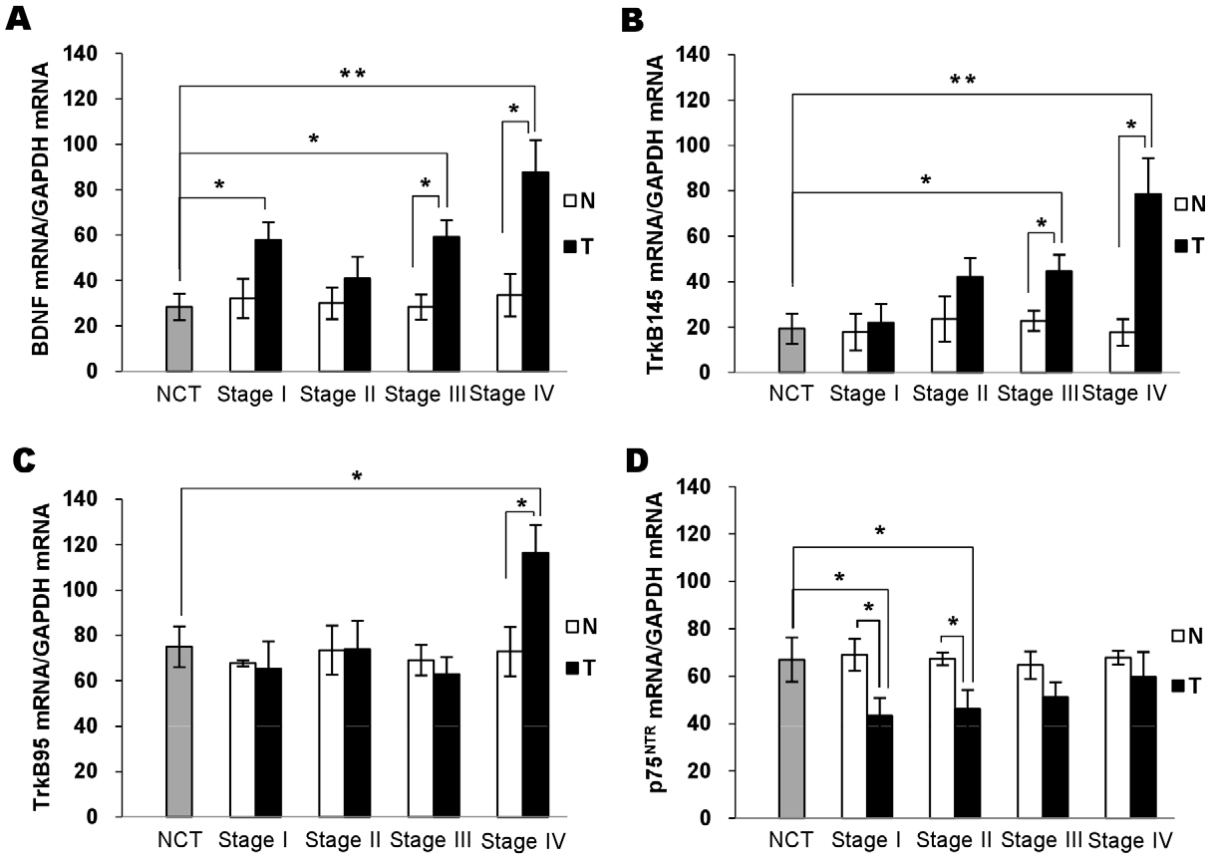
BDNF and its receptors are expressed in colorectal cancer tissues. RT-PCR analysis of BDNF (A), its two high TrkB145 and TrkB95 (B, C, respectively), and low p75^NTR^.(D) affinity receptors in total mRNA extracted from 16 surgically resected primary and metastatic colon adenocarcinoma specimens. *N*, non-tumor tissue (n = 16); *T*, tumor tissue (n = 16); *NCT*, noncancerous tissue (n = 4). Histograms, mean percentage of each amplified mRNA/GAPDH expression of band intensities evaluated by densitometry. Statistical significance:* *p*<0.05; ** *p*<0.01.

BDNF and receptor expression was evaluated by RT-PCR in CRC samples obtained surgically from 16 patients with adenocarcinoma. Samples from patients with benign colorectal disease (megadolichocolon) were studied as controls. Patients' tumor and adjacent non-tumor tissues were compared. BDNF and its receptors TrkB and p75^NTR^ transcripts were detected at the expected size in tissues from patients' colonic carcinoma, whatever TNM stage. BDNF, TrkB145, and TrkB95 expression was higher in tumors than in non-tumor tissues from the same patient and in control (megadolichocolon) tissues (Figure 8A, B and C). Noteworthy, BDNF, TrkB95 and TrkB145 expressions were higher in the advanced CRC stages. In contrast, the expression of p75^NTR^ was lower in tumor samples than in their non-tumor counterparts (Figure 8D). These preliminary data are in accordance with our findings with the cell lines, pointing-to the activation of the BDNF/TrkB machinery in CRC tissues and its likely critical role in tumor growth.

## Discussion

The present data provide evidence that endogenous BDNF is an essential autocrine factor able to rescue human CRC cell death under stressed culture conditions through TrkB, its tyrosine kinase receptor. High BDNF expression was reported in neuroblastoma [39], [40], myeloma [41], B-lymphocytic leukemia cell lines [8], bladder cancer [42], choriocarcinoma [43] or pancreatic cancer [26]. The role of TrkB in tumor invasiveness was first identified in neuroblastoma [39], [40] and its expression is associated with a poor prognosis [40], [44]. Several recent reports focused on TrkB overexpression in various cancers such as ovarian [45], head and neck [46], lung [47], hepatocellular [48], pancreas [49] and bladder carcinomas [42]. Numerous mechanisms underlying TrkB oncogenic function were hypothesized [50]. They were based on epithelial-to-mesenchymal transition in invasive tumors [46], [51], [52] and resistance to anoikis [51], [52], [53]. In addition, BDNF is upregulated by hypoxia through HIF-1α and promotes angiogenesis [19], as described in neuroblastoma models. However, BDNF and TrkB functions in CRC remain unclear despite two studies describing an overexpression of TrkB [29], [30] in CRC tumors. Herein, we provide evidences that an autocrine secretion of BDNF is enhanced by stress culture condition that induces in parallel the relocation of TrkB receptor at the membrane. This mechanism seems to be of major importance in the cancer cell survival through a fine-tuning role of CRC cell growth, as deduced from the proliferative and anti-apoptotic functions of BDNF. It was reported that the BDNF/TrkB pathway promotes cell survival and protects cells from DNA damaging agents in neuroblastomas cells [54], [55]. BDNF increases the proliferation of TrkB-expressing SY5Y neuroblastoma cells, whereas it has no effect on the cells without TrkB expression [39]. BDNF has been shown to enhance the survival of both myeloma [41], [56], [57] and B-cell lines after serum deprivation [8], [58] whereas, anti-BDNF Ab decreased tumor growth in myeloma and neuroblastoma models [57], [59]. Likewise, experiments using blocking anti-BDNF mAb enhanced CRC cell death, since exogenous BDNF increased cell growth and decreased apoptotic ratios of CRC cells in serum-free condition. It was previously shown that BDNF triggers several survival signaling pathways such as PI3-kinase/Akt in neuroblastoma cell lines [60] as well as in embryonic cortical neurons from rats [61]. However, different biological effects induced by BDNF activation were described. Whereas BDNF stimulated the differentiation of normal neural stem cells during brain development depending on neuronal NO synthase activity [62], a proliferative effect was achieved on several tumor cells such as ovarian [63], neuroblastoma [39], myeloma and malignant human B cell lines [41], [56], [57]. By contrast to normal embryonic neural stem cells, such differences in BDNF properties in tumor cells could be a hallmark of tumor invasiveness.

BDNF is synthesized by neurons as pro-BDNF that is cleaved by matrix metalloproteases (MMP), especially MMP-7 and MMP-9 [2] to produce the mature form of BDNF. This process was not previously reported in CRC cells. Interestingly, the expression of these MMP was reported in CRC patients' tissues [64] suggesting that these MMP could be implicated in the cleavage of pro-BDNF in CRC cells.

That BDNF is secreted in association with sortilin, especially under stress conditions, was not previously described in CRC cells. Sortilin was initially known to regulate neurotrophin traffic in human neuronal cells [15] and B lymphocytes [8]. It has been shown in human neuronal cells that sortilin plays a key role in the intracellular transport of neurotrophins and proneurotrophins, which leads to axonal growth and neural cells proliferation [1], [15], [65], [66]. When p75^NTR^ signals independently of Trk, it requires sortilin as a coreceptor, then binds pro-BDNF and induces apoptosis [9], [10], [67], [68]. The present results point for a dual function of sortilin in CRC, the induction of survival, through its trafficking and secretion of mature BDNF and a proapoptotic function through its p75^NTR^-sortilin complex bound to pro-BDNF. Therefore, the role of sortilin seems to be a complex balance between these two opposite functions. Shedding of the luminal domain of sortilin as described in HT29 cells [18] could be another way for CRC cells to prevent the apoptotic effect of pro-BDNF.

Previous reports have shown that the BDNF/TrkB pathway promotes tumorigenesis, invasiveness, angiogenesis and drug resistance, contributing significantly to the aggressive phenotype of these poor prognosis tumors. Thus, an evaluation of BDNF/TrkB expression in patients with CRC disease may be helpful for a better prediction of the prognosis and treatment outcome. In the present study, we showed that BDNF and TrkB (both forms) were overexpressed in tumor tissue in comparison either to each corresponding non-tumor tissue from the same patient or to the control tissues with benign disease. In addition, it was reported that the low affinity receptor p75^NTR^ acts as a tumor suppressor in neuroblastoma cell in vivo [69], and low p75-expressing neuroblasts were detected in primary poorly differentiated neuroblastomas [70]. Our observation concerning the decrease of p75^NTR^ receptor in tumor was consistent with these findings. Our results suggest that BDNF and its receptors may have a crucial function in facilitating tumorigenesis and progression of CRC tumors.

Taken together, all these findings strongly support that autocrine BDNF/TrkB signaling contributes to tumor cell survival in CRC in *vitro* and in *vivo*. We also speculate that sortilin, as a transport protein as well as a potential pro-BDNF receptor, appears to be the key actor of this autocrine loop. Future therapeutic strategies involving BDNF/TrkB and sortilin could be developed from such results to improve targeted treatment of CRC patients.
